# A solitary nodule of Kaposi’s sarcoma

**DOI:** 10.1016/j.jdcr.2024.12.041

**Published:** 2025-03-04

**Authors:** Sanuri Hennayake, Danielle Peterson, Bijan Safai, Andrew Jarad Peranteau

**Affiliations:** Department of Dermatology, New York Medical College, Metropolitan Hospital, New York, New York

**Keywords:** HIV, Kaposi sarcoma

## Introduction

Human immunodeficiency virus (HIV) associated Kaposi’s sarcoma (KS) is decreasing in incidence due to increasing utilization of antiretroviral therapy (ART) among those living with HIV.^1^ Previously seen in those with low CD4 count (<200 cells/mm^3^) and high viral load (>300 copies/mL), recent case studies are increasingly demonstrating the presence of HIV associated KS despite ART, low viral load, and high CD4.^2^, ^3^, ^4^ In this report, we describe a case of KS in a patient with a single nodular papule, a barely detectable HIV viral load, and no other AIDs-defining illness.

## Case report

A 36-year-old non-Hispanic, African American man presented to our clinic with a 2-month history of a nonitchy, nonpainful brown bump on his arm. He denied any traumatic injury. Past medical history was significant for positive status for HIV and patient reported currently taking ART. However, further questioning revealed that the patient had been intermittently inconsistent in taking oral ART early in the year and was transitioning to injections. His viral load had been previously 41 copies per milliliter 4 months prior to presentation but was <20 copies per milliliter at presentation (>300 copies/mL) with a CD4 T cell count of 1235 cells/mm^3^ (500-1500 cells/mm^3^).^2^^,^^3^^,^^5^

**Fig 1:**
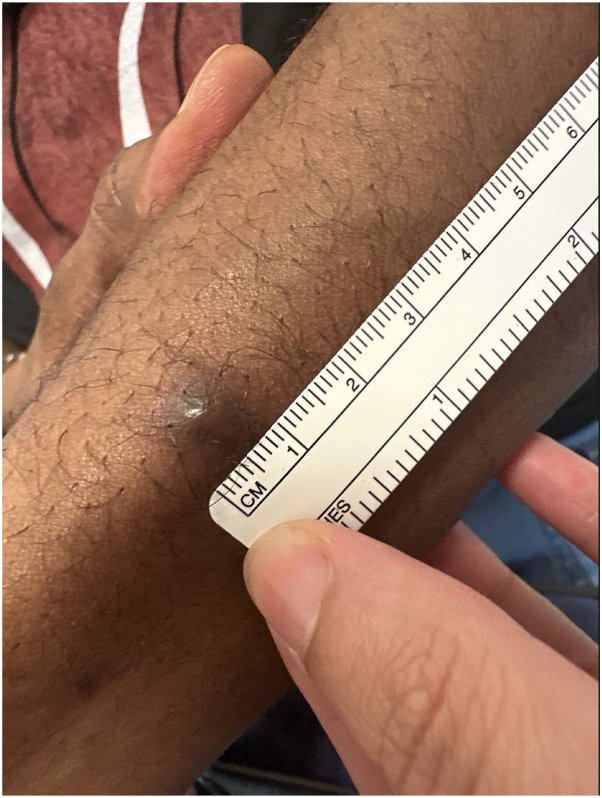
Nine millimeter hyperpigmented firm nodule.

**Fig 2:**
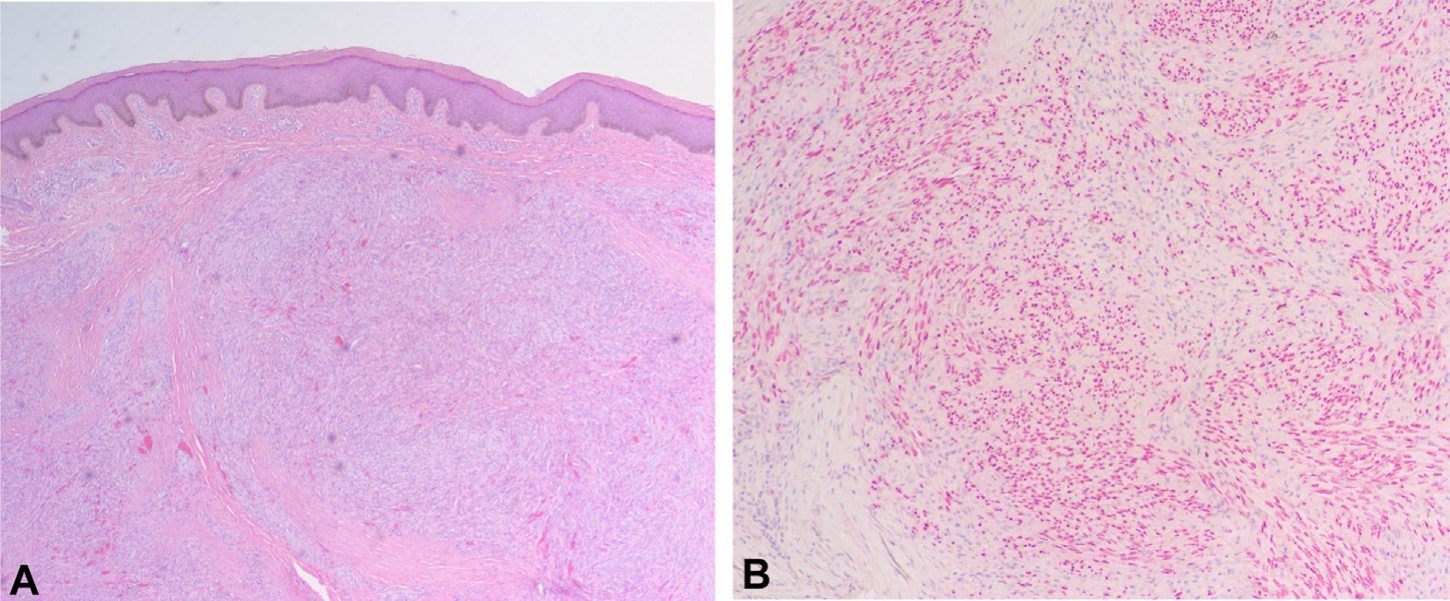
**A,** Punch biopsy demonstrates (**B**) positive HHV-8 stain. *HHV-8*, Human herpes virus 8.

Dermatologic examination of his left dorsal forearm revealed a 9 mm hyperpigmented firm nodule (Fig 1). The lesion was biopsied with a 5 mm punch and sent to pathology. Histology revealed a dermal nodule with spindle cell fascicles with dilated channels and hemorrhages (Fig 2). Immunostaining was positive for human herpes virus 8 which is consistent with KS (Fig 2). Patient returned to clinic for excision of remaining lesion. Viral load at time of complete excision was undetectable and patient presented without any new lesions at 2 week follow up after resection, and remained clear 2 months after initial KS diagnosis. Patient started ART injections 2 weeks after KS diagnosis.

## Discussion

Human herpes virus 8- drives KS, a historically AIDS-defining illness in patients with advanced HIV.^6^ A double-stranded DNA virus, human herpes virus 8 primary infects and maintains life time latency in lymphovascular endothelial and B lymphocytic cells.^7^ Prior to the development of ART, HIV associated KS was seen in patients with CD4 counts less than 1500 cells per cubic milliliter and viral loads of >10,000 copies per milliliter.^3^ As utilization of ART has increased in patients with HIV, an increasing number of cases have been seen in HIV-positive patients with CD4 counts greater than 300 cells/mm^3^ with undetectable viral loads.^2^ Although KS is known as one of the most prominent AIDS-defining illnesses in patients, it is now more frequently diagnosed after the initiation of ART.^8^^,^^9^

Similarly, use of ART has changed the clinical picture of HIV associated KS. HIV-positive patients with a viral load of less than 50 copies for at least 1 year tend to have locally indolent lesions that occur unilaterally (primarily a lower extremity), with less than 5 lesions overall, compared to more viremic patients.^6^

Given the greater number of individuals surviving long term with HIV, some theorize the development of KS despite an adequate CD4 count and low viral load may be related to long term immune system attenuation due to the HIV virus, and protease inhibitors may prevent KS development but their use is limited due to cost and toxicity.^1^^,^^10^ The shift in clinical picture of HIV associated KS must shift clinician’s perspective when encountering HIV positive patients. Regular skin checks, higher index of suspicion for new lesions, and low threshold to biopsy may be warranted in the aging HIV population.

## Conclusion

HIV associated KS is shifting clinically given the aging population of survivors on ART. Here, we present a case of HIV associated KS in an otherwise healthy patient with very low viral load. Ongoing work to investigate the pathophysiology behind the development of HIV associated KS among those on ART therapy is warranted. Diligence amongst dermatologists to recognize and biopsy concerning lesions amongst these patients is critical.

## Conflicts of interest

None disclosed.
